# Socioeconomic Inequalities in Different Types of Disabilities in Iran

**Published:** 2018-03

**Authors:** Ghobad MORADI, Farideh MOSTAFAVI, Mohammad HAJIZADEH, Mohammad AMERZADE, Amjad MOHAMMADI BOLBANABAD, Cyrus ALINIA, Bakhtiar PIROOZI

**Affiliations:** 1. Social Determinants of Health Research Center, Kurdistan University of Medical Sciences, Sanandaj, Iran; 2. Faculty of Health, School of Health Administration, Dalhousie University, Halifax, Nova Scotia, Canada; 3. Dept. of Health Services Management and Economics, School of Public Health, Tehran University of Medical Sciences, Tehran, Iran; 4. Dept. of Public Health, School of Health, Urmia University of Medical Sciences, Urmia, Iran

**Keywords:** Socioeconomic status, Inequalities, Disabilities, Concentration index, Iran

## Abstract

**Background:**

This study measured socioeconomic inequalities in different types of disabilities in Iran. We also examined the prevalence of disabilities across different socio-demographic groups in Iran in 2011.

**Methods:**

This was cross-sectional study using secondary data analysis on all Iranian. Data related to disability prevalence and socioeconomic status (SES) of each province was extracted from the 2011 National Census of Population and Housing (NCPH) and the 2011 Households Income and Expenditure Survey (HIES), conducted by Statistical Center of Iran (SCI). The concentration index and concentration curve were used to measure and illustrate socioeconomic inequalities in different types of disabilities. Chi-squared test was also used to examine the relationship between the socio-demographic variables (age-groups, sex, education level, employment status) and disability.

**Results:**

The results suggested the existence of socioeconomic inequalities in blindness, deafness, vocal disorders and hand disorders in Iran. The concentration index for these four disabilities were −0.0527 (95% confidence interval [CI]: −0.0881, −0.0173), −0.0451 (CI: −0.0747, −0.0156), −0.0663 (CI: −0.1043, −0.0282) and −0.0545 (CI: −0.0940, −0.0151), respectively. There were also significant associations between the demographic variables such as age-groups, sex, education level, employment status and disability (*P*<0.05).

**Conclusion:**

There were significant socioeconomic inequalities in different types of disabilities in Iran with poorer provinces having higher prevalence of disabilities in blindness, deafness, vocal disorders and hand disorders. Strategies to address the higher prevalence of different types of disabilities among poorer provinces should be considered a priority in Iran.

## Introduction

Disability is “complex, dynamic, multidimensional, and contested” (1). According to the International Classification of Functioning, Disability and Health (ICF), people with disabilities have at least one problem in the following three areas: 1) Disorders, defined as any kind of problem or change in body functions, such as paralysis or blindness, 2) Activity limitations, defined as any kind of problem in carrying out activities, such as eating food or walking, and 3) Participation restrictions, defined as any kind of problem in participating in every part of life that leads to discrimination in employment time (1, 2).

According to the World Health Organization (WHO), there were more than one billion people suffered at least one kind of disabilities in 2010 and more than 10 million people were added to this population each year (1). Disabled people in developing countries are generally poor, dependent, unemployed, deprived of formal and professional training, and under oppression and violence. Disability pattern in each country is under the influence of health conditions and socioeconomic factors (3). People with disabilities experience more undesirable socioeconomic consequences than people without disabilities (4). Disability can cause socioeconomic deprivations. The socioeconomic deprivations can also cause or intensify disability (5). Risk factors such as dangerous jobs and lack of suitable and fair access to health services can increase the possibility of encountering with disability or intensify it (6).

The disability influence vulnerable populations disproportionately. Prevalence of disability in low-income countries is more than high-income countries. Moreover, disability is more common among people in the poorest wealth quintile (7).

Low-income, unemployed and illiterate people have higher risk of disability. Poor and ethnic minority children are significantly at greater risk of disability compared to other children (8). Poverty can cause disability by malnutrition, inaccessibility to health services, dangerous living and working conditions. On the other hand, disability can easily cause poverty by losing income, unemployment and additional costs due to disability such as medical costs, housing, and transportation (9). There is even more possibility for families with a disabled member to face catastrophic health expenditures and fall below the poverty line (10, 11). Disabled people and their families experience worse socioeconomic consequences compared with people without disability (2).

In Iran, more than one million people have at least one type of disability. Disabilities in Iran can be classified into several groups. Some of these disabilities are congenital and caused by genetic factors and others are due to traffic and non-traffic accidents (12, 13). Measuring inequalities in the distribution of different types of disabilities can provide valuable information to policymakers, enabling them to identify geographic areas and vulnerable groups for their policy implications. Identifying the disabled community and their distribution in the country can help policy-makers to potentially address some of the factors causing different disabilities in the society (12, 13). Despite the importance of this issue, the distributions of different types of disabilities across different socioeconomic and socio-demographic groups are poorly understood. This study, for the first time, aimed to measure socioeconomic inequalities in different types of inequalities in Iran.

## Materials and Methods

This study used secondary cross-sectional survey datasets in Iran. Data on disabilities were obtained from the 2011 National Census of Population and Housing (NCPH), conducted by Statistical Center of Iran (SCI) (14). Family questionnaire in the NCPH collected information on the disability status of individuals using the following question: “Is there anybody in the family with at least one kind of disabilities such as blindness, deafness, vocal disorders, physical disorders (hand defect or amputation, foot defect or amputation, body defect) or intellectual disorder?”. This section of the questionnaire was completed by each family member. The prevalence of different types of disabilities were calculated for each province, separately. The family questionnaire in the NCPH also collected sex, age, education level and employment status of each family member. The socioeconomic status (SES) of each province was measured based on an assets index computed for each province using the data on assets ownership of households (percent of the households that own computers, washing machines, dishwashers, vacuum cleaners, refrigerators, freezers, fridge freezers, car and Internet) collected in the 2011 Households Income and Expenditure Survey (HIES) (14).Using a principal component analysis (PCA) method (15), an asset index was calculated for each of province and SES of each province has been determined by the assets index of the households computed for each province. Provinces were divided into five SES quintile groups (1 = poorest, 5 = richest) based on the asset index.

The concentration index and concentration curve have been used to compute and illustrate socioeconomic inequalities in disabilities. The convenient covariance approach was used to calculate the concentration index as follows:

Equation (1) $$C=\frac{2}{\mu }\mathrm{cov}\;(y_{i},R_{i}),$$

where *C* indicates the concentration index, *Cov* is the covariance, *y*_*i*_ is the health variable, *R*_*i*_ is the fractional rank for individual *i* in the socioeconomic distribution and *μ* is the mean of health (disability) variable (15). The *C* index ranges between +1 and −1, with zero indicating “perfect equality”. Negative values of the *C* index suggest that disability is concentrated among socioeconomically disadvantaged groups, and *vice versa* (15). Since the outcome variables of interest are binary variables, as per Wagstaff (16), we normalized the index by multiplying by $\frac{1}{1-\mu }$ (i.e., $C_{n}=\frac{C}{1-\mu }$ ). We used a user-written Stata command “conindex” (17) to calculate the *C*_*n*_.

The concentration curve plots the cumulative percentage of health (disability) variable on it y-axis, against and the cumulative percentage of provinces ranked by their SES on the x-axis. In a special case in which each SES quintile of the population, have an equal share of health (disability) variable, the concentration curve would follow the line at 45 degrees (i.e., perfect equality). The concentration curve lies above (below) the line of perfect equality if health (disability) variable is concentrated among the poor (wealthier) people (15). Chi-squared test was used to check the relationship between disability and demographic variables (age, sex, education level and employment status). All the analyses were performed using Stata v13 (Stata Corp, Texas, USA).

## Results

The prevalence of different types of disabilities in each province reported in Table 1. Approximately, 14 per 1000 Iranian had at least one type of disability in 2011. The most common type of disability in Iran was the physical disability (hand defect or amputation, foot defect or amputation and body defect). Southern Khorasan province had the highest prevalence of deafness disability (1.9), vocal disorders (2.8), physical disorders (10.7) and intellectual disorder (5.9) and Hormozgan Province had the highest prevalence of blindness (2.3).

**Table 1:** Prevalence of different types of disabilities in Iran by province in 2011

***Province***	***Prevalence of disabilities per 1000 population***								
**Name**	**Population**	**Average households' size**	**Socioeconomic status quintile (1 = poorest, 5 = richest)**	**Blindness**	**Deafness**	**Vocal**	Physical^*^	**Intellectual**	**Any type**
Ardabil	1248488	3.8	1	1.4	1.6	2.2	7.1	4.7	13.6
Kohgiluye and Boyer-Ahmad	537411	4.9	1	1.7	1.7	2.8	9.4	4.7	17.5
Golestan	1575443	3.8	1	1.4	1.4	2.2	10.4	5.1	17.7
Hamedan	1598346	3.5	1	1.5	1.5	1.8	9.0	4.6	16.0
Ilam	503840	4.7	1	1.5	1.6	2.3	8.6	4.8	16.0
Kerman	2603563	3.9	1	1.4	1.2	1.6	6.9	4.0	13.1
Kermanshah	1667735	3.9	1	1.7	1.7	2.2	9.5	4.6	17.4
Khuzestan	4531720	4.4	1	1.8	1.4	2.0	8.3	4.6	14.5
Kurdistan	1346680	3.6	1	1.6	1.5	2.2	9.9	4.6	16.7
Lorestan	1580284	4.4	1	1.6	1.5	1.9	8.4	4.4	15.6
Sistan and Baluchistan	2102222	3.8	1	1.6	1.1	1.8	5.9	3.8	12.6
South Khorasan	585212	3.7	1	1.7	1.9	2.8	10.7	5.9	19.3
North Khorasan	710315	3.6	1	1.3	1.7	2.4	9.2	5.3	17.8
Hormozgan	1376964	4.3	1	2.3	1.3	1.8	6.7	3.6	13.8
Qazvin	1087436	3.7	2	1.2	1.3	1.6	6.4	3.9	12.8
Razavi Khorasan	5335442	3.6	2	1.3	1.4	2.0	8.8	5.3	16.0
East Azerbaijan	3724620	3.5	2	1.1	1.3	1.5	6.2	4.1	11.7
Zanjan	915289	3.7	2	1.3	1.3	1.7	7.2	3.9	13.7
Semnan	574977	3.4	2	1.0	1.1	1.5	6.3	4.1	12.5
West Azerbaijan	2752206	3.9	2	1.2	1.2	1.8	7.9	4.1	13.9
Markazi	1287988	3.6	3	1.4	1.4	1.8	8.6	4.6	15.8
Alborz	2064582	3.5	3	0.9	1.0	1.3	6.2	3.5	11.5
Gilan	2307732	3.3	3	1.1	1.5	2.5	9.5	5.1	16.2
Charmahale-Bakhtiari	741486	3.8	3	1.5	1.5	2.0	9.2	5.2	17.8
Qom	1151672	4.0	4	1.4	1.4	1.5	8.5	4.7	14.3
Yazd	954093	3.4	4	1.5	1.4	1.8	9.2	4.6	16.4
Fars	4159665	3.8	4	1.6	1.7	2.4	9.6	5.1	17.0
Boshehr	918044	4.2	4	1.4	1.2	2.0	7.0	4.2	12.3
Esfahan	4450808	3.4	5	1.3	1.4	1.8	9.1	5.2	16.3
Mazandaran	2833680	3.4	5	1.3	1.4	2.2	8.9	4.6	15.2
Tehran	12183391	3.4	5	0.9	1.0	1.2	6.6	3.7	10.9
**Iran (all Provinces)**	75149669	3.7	-	1.3	1.3	1.8	8.0	4.4	13.5

* This includes hand defect or amputation, foot defect or amputation and body defect.

The prevalence of disability by sex is presented in Table 2. The prevalence of disability was more than their counterparts (*P*<0.05). The prevalence of disability was higher among older age-groups (*P*<0.05) (Table 2). The prevalence of disability was significantly higher among illiterate people compared to other educational groups (*P-value*<0.05). The prevalence of disability was significantly lower among employed people than unemployed people (*P*<0.05).

**Table 2:** Prevalence of disability by sex, age-groups, education level and employment status in Iran, 2011

***Variables***		***Disability***				
		**Yes**		**No**		***χ*^2^*(P-value)***
		**Number**	**Percentage**	**Number**	**Percentage**	
Sex	Male	216777	2.0	10636440	98.0	7258.9 (0.001)
	Female	131093	1.2	10462473	98.8	
Age-groups(yr)	0–4	22071	0.4	6210481	99.6	
	5–14	104189	0.9	11225037	99.1	
	15–24	174149	1.2	14847391	98.8	331788.6 (0.001)
	25–34	194672	1.2	15449906	98.8	
	35–44	151512	1.4	10326255	98.6	
	45–54	125703	1.7	7432186	98.3	
	55–64	78572	1.7	4464454	98.3	
	65–74	69207	2.8	2394492	97.2	
	75+	97126	5.3	1735944	94.7	
Level of educational attainment (6 years and above)	Illiterate	460865	4.7	9258847	95.3	
	Junior high school and lower	353792	1.2	29655880	98.8	884960.3 (0.001)
	High school	125408	0.7	17321954	99.3	
	College and university	47657	0.5	10457473	99.5	
Employment status (10 years and above)	Employed	191982	0.9	20354892	99.1	7582.9 (0.001)
	Unemployed	51042	1.4	3508976	98.6	

The *C* index suggested the existence of socioeconomic inequalities in blindness, deafness, vocal disorders and hand disorders in Iran. The concentration index for these four disabilities were −0.0527 (95% confidence interval [CI]: −0.0881, −0.0173), −0.0451 (CI: −0.0747, −0.0156), −0.0663 (CI: −0.1043, −0.0282) and −0.0545 (CI: −0.0940, −0.0151), respectively. Although the *C* index indicated that other types of disabilities were concentrated among socioeconomically poor provinces, these results were not statistically significant at 95% significance level (Table 3).

**Table 3:** The concentration index for different types of disabilities in Iran, 2011

***Disability***	***Concentration index***	***Standard Error***	***95% Confidence interval***	***P-value***
Blindness	−0.0527	0.0173	−0.0881, −0.0173	0.005
Deafness	−0.0451	0.0144	−0.0747, −0.0156	0.004
Vocal disorders	−0.0663	0.0186	−0.1043, −0.0282	0.001
Amputation of hand	−0.0434	0.0218	−0.0881, 0.0012	0.056
Hand disorders	−0.0545	0.0193	−0.0940, −0.0151	0.008
Amputation of leg	−0.0137	0.0164	−0.0472, 0.0199	0.142
Leg disorders	−0.0144	0.0192	−0.0537, 0.0248	0.458
Body disorders	−0.0135	0.0210	−0.0564, 0.0293	0.524
Intellectual disorders	−0.0144	0.0134	−0.0418, 0.0129	0.289
Total (at least with one disability)	−0.0239	0.0132	−0.0509, 0.0030	0.080

**Fig. 1:**
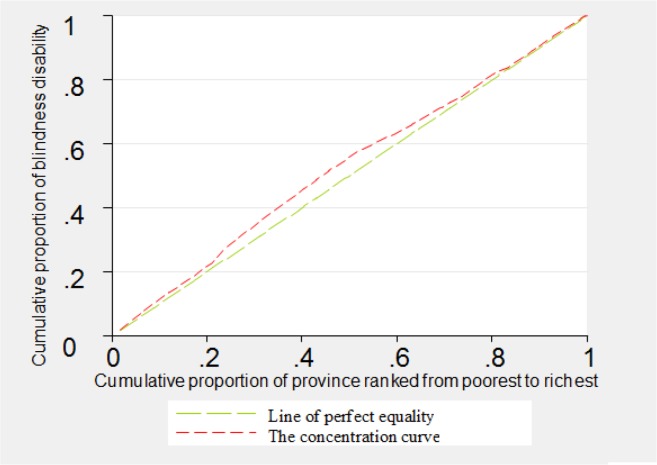
The concentration curve for blindness in Iran, 2011

**Fig. 2:**
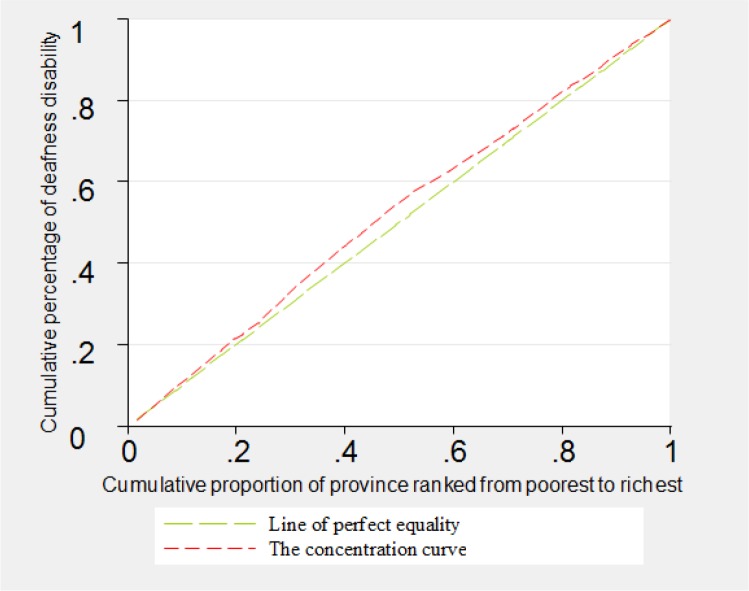
The concentration curve for deafness in Iran, 2011

**Fig. 3:**
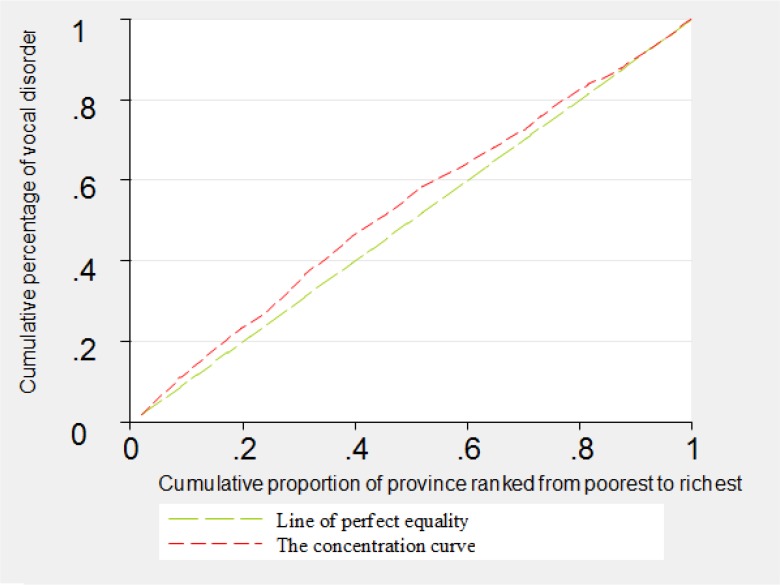
The concentration curve for vocal disorder in Iran, 2011

**Fig. 4:**
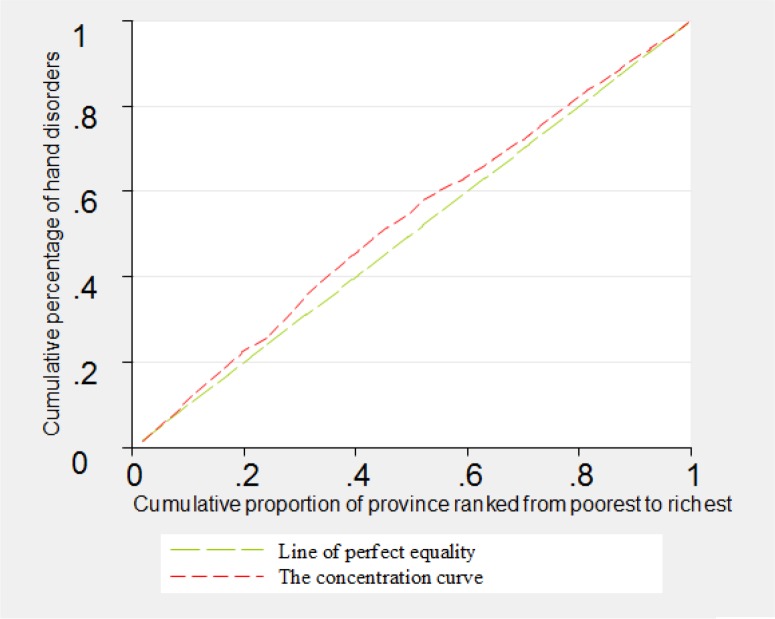
The concentration curve for hand disorder in Iran, 2011

The concentration curves related to disabilities of blindness, deafness, vocal disorder and hand disorders were shown in Figs. 1–4. As can be seen, the concentration curve for all of the four aforementioned disabilities lied above the line of perfect equality, suggesting the concentration of these disabilities among the poorer province.

## Discussion

This study aimed to measure the prevalence of different types of disabilities across different socio-demographic groups and measure socioeconomic inequalities in different disabilities in Iran. The results revealed that 13.5 people per 1000 population had at least one type of disabilities in Iran. South Khorasan province had the highest prevalence of disabilities (deafness, physical, intellectual and at least one kind of disability) among 31 provinces of Iran.

The results of the *C* index showed that blindness, deafness, vocal disorder, hand disorders were more common among poorer provinces. Higher prevalence of disability among lower SES groups was in line with the findings of previous studies in Iran and elsewhere. For example, a study by Entezarmahdi and colleagues showed that disability caused by leprosy is more concentrated among families with lower SES (18). The greater risk of disability was also observed among poorest SES quintiles in other developing countries (19, 20). Poverty and disability have a two-way relationship, so that not only poverty could increase the probability of disability but also disability increases the risk of falling into poverty (2, 20, 21).

Our findings also showed that the prevalence of disability in working people was less than unemployed. Unemployment rate for people with disabilities in Iran was twice as much as that rate for people without disabilities. The difference in the employment rates between people with and without disabilities has been documented in other countries. For example, this gap in South Africa, Japan, Switzerland, and Malawi was reported 30%, 38%, 81%, and 92%, respectively (1). A survey conducted by the WHO in 51 countries, showed that the average employment rate for disabled males and females, was 52.8% and 19.6%, respectively; however, this rate for male and female without disabilities was 64.9% and 29.9%, respectively (22).

The results of this study also showed the highest percentage of disability among the elderly population in Iran. One of the reasons for the higher disability rate among older population is that older people compared to their younger counterparts have more physical disabilities (3, 23, 24). The results also showed that the percentage of disability among the illiterate is more than others educational groups. According to the WHO report on disability, disabled people have less and unequal access to education in comparison with others (25); thus, they have worse educational attainment level (26, 27).

Similar to a study by Entezarmahdi and colleagues (18), the results of this study also showed that the percentage of disability among men was more than women. This result is consistent with a study in 59 countries which reported a higher prevalence of disability among women compared to men (28). Gender inequalities in disabilities can be explained by the variety of lifestyle factors. For example, most women in Iran are homemakers and therefore are at lower risk of work-related disability.

This study is subject to two main limitations and the findings should be interpreted with caution. *First*, since this study was drawn from cross-sectional datasets, causality cannot be inferred. *Second*, due to the availability of data we used provincial level SES data to measure socioeconomic inequalities in disabilities. Using provincial level data captured between-province variation in disabilities but ignored within-province variation in disabilities.

## Conclusion

This study demonstrated that poorer provinces in Iran having the higher prevalence of disabilities such as blindness, deafness, vocal disorders and hand disorders. Strategies to address the higher prevalence of different types of disabilities among poorer provinces should be considered a priority in Iran. As disability is a development issue due to its bilateral relationship to poverty, disability prevention programs and rehabilitation programs should be targeted towards high-risk population and provinces with the highest prevalence of disabilities in Iran.

## Ethical considerations

Ethical issues (Including plagiarism, informed consent, misconduct, data fabrication and/or falsification, double publication and/or submission, redundancy, etc.) have been completely observed by the authors.
